# Upper Limb Phlegmasia Cerulea Dolens Secondary to Heparin-induced Thrombocytopenia Leading to Gangrene

**DOI:** 10.7759/cureus.2853

**Published:** 2018-06-21

**Authors:** Anuhya Kommalapati, Avyakta Kallam, Jairam Krishnamurthy, Sri H Tella, Jahnavi Koppala, Pavan Kumar Tandra

**Affiliations:** 1 Internal Medicine, University of South Carolina, Columbia, USA; 2 Hematology and Oncology, University of Nebrask, Omaha, USA; 3 Hematology Oncology, University of Nebraska Medical Centre, Omaha, USA; 4 Internal Medicine, Creighton University, Omaha, USA; 5 Hematology and Oncology, University of Nebraska, Omaha, USA

**Keywords:** heparin-induced thrombocytopenia, phlegmasia, hemodialysis, sepsis, gangrene, argatroban

## Abstract

We present a case of a dialysis-dependent end-stage renal disease patient who originally presented with sepsis and later developed heparin-induced thrombocytopenia-related upper extremity deep venous thrombosis that rapidly progressed to phlegmasia. Argatroban, a direct thrombin inhibitor, was initiated without delay. Argatroban restored the venous patency completely but did not reverse his two gangrenous fingers. The patient finally underwent digital amputation. The management of this uncommon, but life-threatening, situation of upper limb phlegmasia cerulea dolens secondary to heparin-induced thrombocytopenia leading to gangrene is discussed in this case report.

## Introduction

Heparin-induced thrombocytopenia type II is a well-known non-hemorrhagic complication of heparin therapy, which is caused by immunoglobulin G (IgG) antibodies directed against the heparin-platelet factor 4 complexes [1]. In patients who are undergoing dialysis, the prevalence of heparin-induced thrombocytopenia was reported to be 0.26% with the majority of patients using unfractionated heparin rather than low-molecular weight heparin [2]. However, the increased use of central venous catheters and the liberal use of venous Doppler have contributed to the increased incidence of upper extremity deep venous thrombosis in dialysis patients. The deep venous thrombosis may proceed to an uncommon and severe form of thrombosis called phlegmasia cerulea dolens [3-4]. It is a severe form of venous thrombosis leading to the occlusion of major and collateral veins, thereby causing painful swelling, cyanosis, and edema of the affected limb. Upper extremity deep venous thrombosis leading to phlegmasia cerulea dolens occurs in approximately 2%-5% of these cases. Phlegmasia may even develop into gangrene by impairing arterial inflow [5] and approximately half of the patients end up having an amputation of the associated extremity [6].

## Case presentation

**Figure 1:**
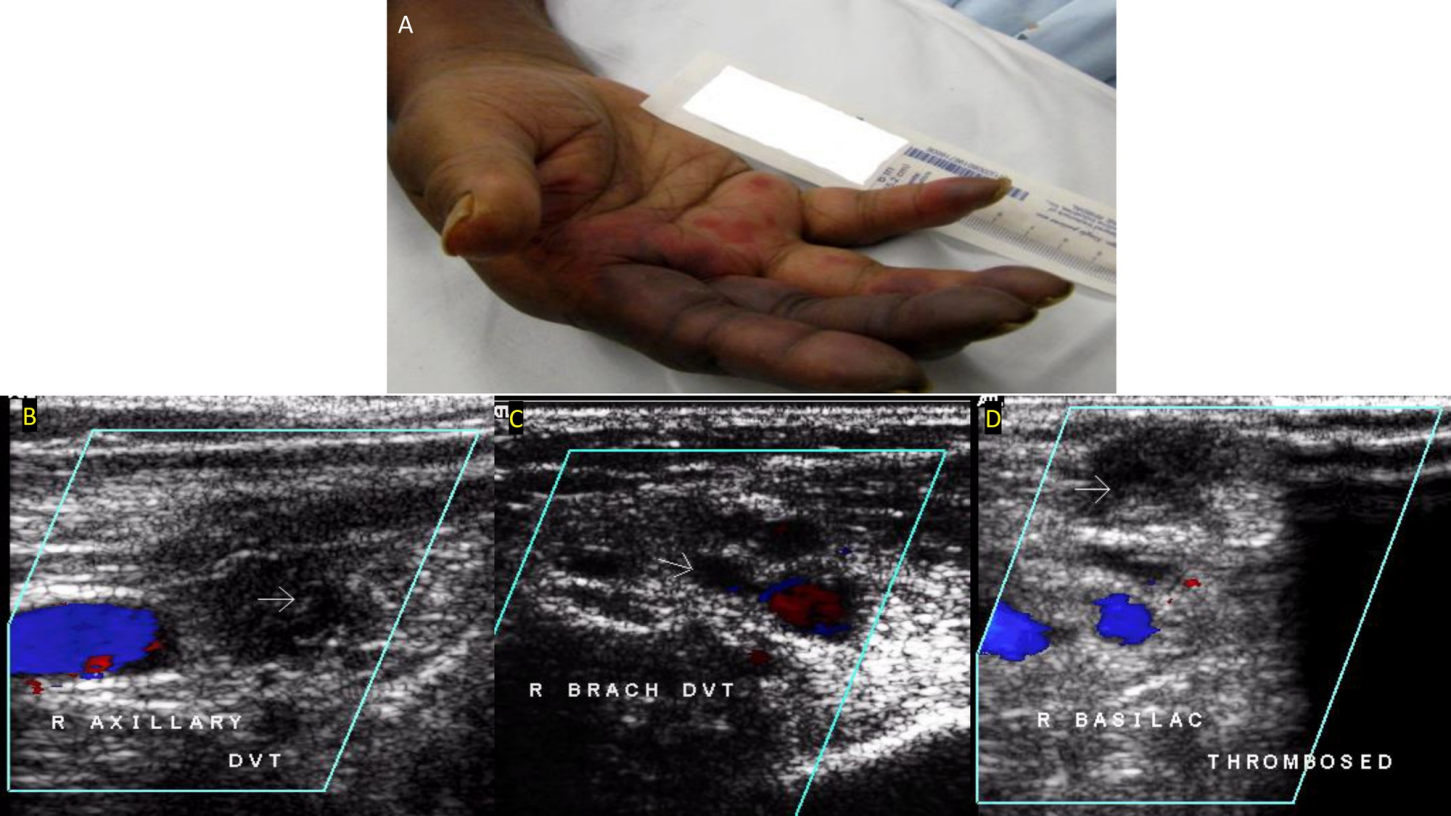
A) Right-hand gangrene; B) Venous duplex showing deep venous thrombosis in the right axillary vein; C) Venous duplex showing deep venous thrombosis in the right brachial vein; D) Venous duplex showing deep venous thrombosis in the right basilica veins

An 88-year-old man was admitted to our facility with altered mental status, hypotension (blood pressure range: 71-84/47-57 mmHg in the right arm supine position), fever (104^o^F), and tachycardia (heart rate: 140-150 beats/min) on arrival at the emergency room. Prior to admission, he was on hemodialysis for the past three months for end-stage renal disease secondary to rapidly progressive glomerulonephritis (has a right permacath). He was receiving intermittent heparin flushes along with dialysis to maintain the patency of the extracorporeal circuit. Other significant past medical history included a splenectomy in 2007. Clinical manifestations, imaging tests, and blood cultures suggested septic shock secondary to Streptococcal pneumonia. The patient was started on meropenem and vancomycin. A left internal jugular catheter and arterial line (in the right upper extremity) were placed for fluid resuscitation and blood pressure monitoring, respectively, and the patient was managed per surviving sepsis guidelines. On day three of hospitalization, the patient started to complain about a right-hand pain at the site of the arterial catheter. The physical examination was remarkable for a swollen and cyanotic right upper extremity, especially the second and third fingers (Figure 1A), with a barely palpable radial pulse compared to the left side. Arterial Doppler of the upper extremities was obtained, with findings indicative of significant right-sided arterial insufficiency. Further evaluation by venous duplex ultrasound identified a massive thrombus in the axillary, brachial, and basilic veins of the right arm with the solely spared ulna vein being hugely engorged (Figures 1B-1D).

We considered the possibility of catheter-induced venous thrombosis, sepsis-associated disseminated intravascular coagulation and heparin-induced thrombocytopenia as working diagnoses. Given the suspicion of heparin-induced thrombocytopenia, we discontinued heparin immediately while the patient’s peripheral smear and coagulation cascade were investigated. Although the patient had low platelets (Table 1), prolonged prothrombin time (16 (normal: 11-13 sec)), activated partial thromboplastin time (63 (normal: 25-35 sec)), elevated fibrin degradation products (>40 (normal: <10 mcg/ml)), which were suspicious for sepsis-related disseminated intravascular coagulation; normal  factor VII (77 (normal: 50-150%)), high VIII levels (192 (normal: 50-150%)), normal haptoglobin (163 (normal: 36-195 mg/dl)), mildly decreased hemoglobulin (range: 12.1-12.9 (normal:13.5-17.5 g/dl)), and the presence of very few schistiocytes (<0.5%) on a peripheral smear made disseminated intravascular coagulation less likely. Laboratory data were remarkable for a significant drop in the platelet count from 234*10^9^/L (on initiation of hemodialysis) to 44*10^9^/L (the day of hospital admission) over the past three months. Based on the 4T score, heparin-induced thrombocytopenia was highly suspected and argatroban was initiated at 1 mg/kg/min. Vascular surgery consultation was obtained to address the gangrene in the second and third digits of the right upper extremity. However, considering his critically septic situation with multiple morbidities, a decision was made not to proceed with invasive maneuvers. On hospital day four, heparin-platelet factor 4 (PF4) antibodies (1.67 (normal: <0.4), heparin inhibition: >50%), and serotonin release assays returned positive (using enzyme-linked immunosorbent assay), confirming heparin-induced thrombocytopenia. On hospital day eight, repeated venous duplex demonstrated normal compressibility and spontaneous flow in the vein of the right upper extremity. The platelet count recovered and no further thrombotic complications were observed. Though we were able to manage the patient’s advanced gangrene with argatroban, his second and third fingers were amputated in the end.

**Table 1:** Treatment course with correspondent platelet profile

Event	Hemodialysis		Hospital admission		Argatroban				
Day of treatment	0	64	107	108	109	110	111	112	113
Platelet count (x 10^9^/L)	234	95	44	25	13	12	19	66	75

## Discussion

The report illustrates the diagnostic and therapeutic challenges of heparin-induced thrombocytopenia-related phlegmasia and emphasizes the need for a timely diagnosis to prevent catastrophic complications, as seen in the patient.

Heparin-induced thrombocytopenia type II classically presents five to 14 days after the initiation of heparin therapy but delayed-onset heparin-induced thrombocytopenia has been described in the literature. The time of onset for patients on hemodialysis is usually one to two months with only 20% of patients being diagnosed within five to 10 days of starting dialysis [7]. It should be noted that it is the relative drop in the platelet count as compared to that of the baseline (at least a 30%-50% drop) rather than absolute thrombocytopenia that helps make the diagnosis of heparin-induced thrombocytopenia. Though most patients with heparin-induced thrombocytopenia have a platelet count less than 150*10^9^/L, it has been reported that 10%-15% of patients may have platelet counts more than 150*10^9^/L [8]. Accordingly, the patient might have developed heparin-induced thrombocytopenia much earlier (platelet count dropped from 234*10^9^/L on hemodialysis day one to 95*10^9^/L on day 64).

A good understanding and the appropriate application of other available laboratory tests for heparin-induced thrombocytopenic thrombosis is necessary, as the acute-onset devastating phlegmasia in our patient could also be explained by sepsis-induced disseminated intravascular coagulation and/or central venous catheter-triggered deep venous thrombosis [9]. The most accurate test for identifying heparin-induced thrombocytopenia antibodies is the 14C (radiolabeled) serotonin-release assay (SRA); however, it is time-consuming and highly dependent on the reactivity of the donor’s platelets. The platelet aggregation test in comparison takes less time to perform, but its results vary widely depending on the heparin concentration used and the variability of donor platelets as well. An enzyme-linked immunosorbent assay (ELISA) for heparin/PF4 is another option, easily performed and generally in agreement with serotonin release assay results. However, because of its high false-positive rate, it has become the most common laboratory test to exclude heparin-induced thrombocytopenic thrombosis. Finally, heparin-PF4 rapid assay (PIFA) has also begun to be available at some institutions across the United States. The heparin-PF4 rapid assay is a single-use, single-patient test that can determine a patient’s antibody status within 10 minutes [10]. Nonetheless, it is important to note that not all heparin-induced thrombocytopenia-antibody positive patients may develop heparin-induced thrombocytopenic thrombosis.

Although disseminated intravascular coagulation was excluded retrospectively and the high likelihood of heparin-induced thrombocytopenic thrombosis (based on the 4T score) dissuaded us from taking away the patient’s dialysis catheter, both sepsis and catheter played a key role in his clinical picture. A systemic inflammation caused by bacteremia has been shown to activate the coagulation status by generating tissue-factor-mediated thrombin and downregulating the physiological anticoagulant mechanism [11-12]. In addition, Hong et al have demonstrated that the incidence of upper extremity deep venous thrombosis was significantly higher in heparin-induced thrombocytopenia patients with a central venous catheter than without a central venous catheter (14/145 versus 0/115, p<0.001), while the difference didn’t exist for lower extremity deep venous thrombosis [5]. Local tissue injury by the central venous catheter and the gradual onset of heparin-induced thrombocytopenia complicated by rapidly developing sepsis may explain the patient’s clinical scenario more comprehensively.

Finally, the management of heparin-induced thrombocytopenia thrombosis has been well-established by discontinuing heparin and initiating alternative anticoagulation, direct thrombin inhibitors, such as argatroban or fondaparinux, followed by the transition to warfarin, which has been proved effective [13]. In comparison, the development of a standard treatment for phlegmasia cerulea dolens is still ongoing, with multimodal interventions favored to prevent gangrene. These include systematic or catheter-induced, surgical or interventional venous thrombectomy and the occasional use of an inferior vena cava filter and fasciotomy [14-16]. There are a few case reports of phlegmasia associated with heparin-induced thrombocytopenia type II, but none were reported in patients with end-stage renal disease [17-18]. Fondaparinux has been successfully used in other clinical scenarios in the management of heparin-induced thrombocytopenic thrombosis [18]. We did not opt for fondaparinux, as it is not indicated in patients with creatinine clearance less than 30 ml/min. In this case, we used appropriately dosed argatroban (metabolized through the liver) without pursuing further interventions due to the concern of introducing additional risks for such a senior, critically ill patient with multiple co-morbidities. Argatroban did successfully resolve the patient’s upper extremity deep venous thrombosis and prevent thrombotic recurrence. Besides, systemic thrombolysis theoretically should reach microcirculatory thrombosis more efficiently than any invasive intervention, as in the case of our patient with mainly two fingers affected. In retrospect, catheter-induced local thrombolysis might be a good adjunctive to save the patients’ fingers, but more studies are needed to evaluate any added benefit versus risk.

After stopping heparin administration in patients with heparin-induced thrombocytopenia, the median time to achieve a platelet count of >150,000 per microliter is about four days. However, in patients with more severe heparin-induced thrombocytopenia as in the present case, the platelet count can take longer to recover. However, it is also important to consider that the patient may also have other concurrent medical problems, such as sepsis or antibiotics usage, contributing to thrombocytopenia.

## Conclusions

Although phlegmasia appears to be rare, it represents a potentially severe complication of heparin-induced thrombocytopenia thrombosis. Our report demonstrates the clinical significance of the early identification of heparin-induced thrombocytopenic thrombosis, which has the potential to be severe and detrimental in dialysis patients who have intravenous catheters in place. This warrants the early initiation of non-renally metabolized antithrombotic therapy, such as argatroban, and the consideration of appropriate screening in patients exposed to heparin who are dropping platelet counts.
